# Elongating the role of renewable energy and sustainable foreign direct investment on environmental degradation

**DOI:** 10.1016/j.heliyon.2023.e18421

**Published:** 2023-07-19

**Authors:** Sobia Naseem, Xuhua Hu, Muhammad Mohsin

**Affiliations:** a School of Finance and Economics, Jiangsu University, Zhenjiang, 212013, Jiangsu, PR China; b Institute of Industrial Economics, Jiangsu University, Zhenjiang, 212013, Jiangsu, PR China; c Business School, Hunan University of Humanities, Science and Technology, Loudi, 417000, Hunan Province, PR China

**Keywords:** GHG emissions, Renewable energy, FDI, DOLS, G-8 countries

## Abstract

Climatic variations and GHG emissions are the most debated issues of the current age economically, socially, politically and environmentally. An internationally legally binding treaty on climate change, the “Paris Agreement” is followed by G-8 countries to maintain environmental sustainability with green development. The research investigates the relationship of GHG emissions with renewable energy (RE), foreign direct investment (FDI), total population (TP), and trade (TR). The time span of 22 years is used for analytical purposes covering the period from 2000 to 2021 b y addressing the literary gap. The analytical procession found total population and trade increase GHG emissions because of its modern fundamental layers toxic human activities and polluted trade practices. The decreasing behavior toward GHG emissions has been determined by FDI and RE. The findings of this research have confirmed the long-run relationship among variables. They are evidence that the eco-innovative steps by G-8 countries significantly reduce GHG emissions directly or indirectly. Furthermore, the analytical outcomes indicate that innovative green development in renewable energy sector can reduce the GHG emissions pressure from this sector and contribute to net zero emissions. The extracting results have suggested policies for environmental practitioners and economic developers.

## 1 Introduction

The greenhouse gas emissions, climate crisis and stratosphere ozone depletion are burning issues that influencing the entire global village. The toxic greenhouse gas emissions generally cause global warming, and consequently, the stratosphere ozone depletion damages the ozone layers by preventing harmful ultraviolet (UV) rays. The harmful ultraviolet (UV) rays directly affect living organisms and the earth's surface [[1], [2], [3]]. The industrial revolution of the 1760s has uplifted the graph of economic growth globally, but the higher pace of economic growth was achieved at the cost of environmental quality compromise [2]. Non-renewable energy resources were used to run industries like coal and fossil fuels [4,5]. The current decade's prevalent challenge is maintaining the balance between economic growth and carbon emission reduction [6]. Foreign direct investment is a critical player in economic growth [7]. FDI is equally important for developing and developed countries to boost the scale of development, and continuous efforts and strategies are designed to attract FDI [8]. FDI helps to open a window toward the world and explore the channels of economic growth. According to statistics for 2019, the Chinese economy has placed itself at the second position in the global list of attractions and usage of FDI amounted to 141.23 billion US dollars. FDI's negative impact on China's environmental quality is also observed. The local government compromises the environmental standards to attract maximum FDI, and the denouement of this growth becomes the reason for environmental pollution with toxic emissions. This heavy dependence on non-renewable energy resources has altered the direction of the economic growth graph and changed the direction of environmental quality [9,10]. The non-renewable energy resources were cheap, reduced capital burden and brought high profits. The strong connection of economic growth, energy consumption and environment are indulged in triumvirate complex structures which the energy transition can resolve.

Non-renewable energy resources should be replaced with renewable energy resources [11,12] to preserve the environmental quality, economic growth and smooth survival of earth's habitats. This is the technology age, and technology's evolutionary contribution cannot be denied. Information and communication technology is proving itself to be an environment-friendly factor with rapid growth strategies of economies. The travel substitute, transport, physical products and many more innovatory contributions of technology make it possible to reduce GHG emissions. People's knowledge about environmental safety awareness can help achieve environmental improvement. The use of green technology to obtain maximum output in perspective of environmental quality is required to determine which parts of society are interlinked with highly emitted activities [13,14]. The strategic planning and decision-making process are based on analyzing total emissions from this sector and comparing the sectorial GHG emission contribution with total GHG emissions so that a cyclic reduction process can be planned. Digital trade can be divided into three kinds of effect orders that relate to emissions and energy consumption [15]. Firstly, the digital hardware of intelligent transport systems consumes energy. Secondly, energy can be saved by using ITS to transport services provided by digital applications. Thirdly and lastly order effect of digital trade is related to the emerging impact of technology in economic development. Third order effect has long-term benefits to enhance efficiency by inducing ITS applications [16,17].

### 1.1 Research significance & contribution

The individuality and contribution of this research in existing literature can be justified with different perspectives. This research approached G-8 countries as a sample because these countries are covered almost 40% of the global economy with 25% of global GHG emissions excluding Russia so far [18,19]. The G-8 countries are industrial-based top tier largest high-income countries. The ratio between economic growth and GHG emissions is lower but not at net zero. Researchers usually give the margin to G-8 countries due to their balanced ratio between economic growth and GHG emissions. At the same time, it's not an ignorable fact because 25% of global emissions are a more significant number for the destruction of environmental quality. Being industrial-based countries, G8 countries are reliant on fossil fuels due to insufficient renewable energy resources, high energy prices, and uncertainties of resources for the future are reducing the level of environmental sustainability and reliability. The green trade, people's awareness FDI halo hypothesis, and renewable energy resources have the potential to simultaneously uplift the graph of economic growth and mitigate environmental deterioration. The G8 nations are working on cutting down GHG emissions to half by 2050 [20], which will improve economic efficiency, social standard, and technical advancement via FDI and international trade. The focus of previous research was human consumption. Still, this research will fill the gap by proposing novelty research about the Heaven & Halo hypothesis by covering energy resources, population, and trade's impact on the fluctuation of GHG emissions in G8 countries. This research approached the maximum possible data to check the real impact and contribute to guiding the environmentalist to reduce emissions by focusing on the most emitted sectors of the economy. The variables of analytical research are also selected with keen interest, with maximum coverage of modern economies, as most countries' energy production is still based on traditional energy resources and few numbers of sectors trying to replace it with renewable energy resources. Population, trade and FDI are equally crucial for individual countries, and their contribution to economic growth and the environment is somehow similar everywhere, whether a developed or developing country. Renewable energy has justified its existence with a reduction in GHG emissions compared to oldfangled energy resources of development. The econometric methods are selected based on the study's nature which will clear the direction of individual sectors toward GHG emissions and development of G-8 countries. The short and long-run relationship among variables, effects of external shocks and reduction of GHG emissions with a high development rate are explained. This research helps the individual sector of G-8 countries and the rest of the world who want to explore the environmental conditions and contribution of sectorial growth with slow-poisoned development methods.

### 1.2 Theoretical background

This research is based on two theories, i.e., comparative advantage and International Product Life Cycle (IPLC). The comparative advantage theory declares that trade is equally beneficial for both parties, which helps to approach for best quality goods at a lower cost [21]. The lower opportunity cost of high-quality products enhances resource management efficiency. The international product life cycle (IPLC) theory explains how an innovative domestic product approaches an international market and enhances the country's export [22]. Leamer & Levinsohn [23] elucidated by applying Ricardian and the Ricardo–Viner models that technological difference is a source of international comparative advantage. The Ricardian model had some conditions: one input (labor) assumed a mobile sector within the country but immobile internationally. The Ricardo–Viner model comprehends this model with two additional factors (sector-specified). This addition twists the production possibility curve, allowing international commerce to influence income distribution. Economic isolation due to technological differences was observed and considered a natural consequence of economic liberalization. Heckscher–Ohlin-Vanek (HOV) model is used for cross-country comparison and is considered an important theorem in international trade. Caron et al. [24] reconsidered the HOV model and explained that the prediction and actual world trade have differences. The trading model of HOV narrated that the trade among rich countries (Developed to Developed) countries is higher than between Rich to poor (developed to developing) countries. While the reason behind the trade ratio is not developed or developing countries, it has based on skilled and proficient labor, which helps to restructure the trade puzzles. The higher trade-to-GDP ratio in high-income countries also comes from the positively correlated relationship between income elasticity and sectors' traceability compared to low-income countries. The HOV model also not including the market for purchasing environmental services, and the cost of environmental appropriation is based on biocapacity, not related to labor and capital first. The HOV model and theorem are perfect for a competitive market, whereas marginal productivity can consider for the global environment because of its absorptive capacity and consistent ecosystem.

## 2 Literature review

The environmental awareness and focus on achieving the target of sustainable development goals (SDGs) grab the attention of the whole world. The recent competition isn't based on a country's industrial development, economic growth, or high per capita income but on how efficiently they maintain their environmental sustainability with achieving the highest financial status globally [25,26]. Accomplishing sustainable development goals (SDGs) is becoming critical for policymakers [27,28]. The SDGs agenda is designed to cover almost all major and important issues of the globe about pollution and basic human needs. Researchers are doing their best to contribute and mark the polluted areas of ecological systems. The graph (Upward & downward) of environmental sustainability and accurate polluted areas identification can help to work toward the right path and achieve the maximum possible stable & sustainable environment. Energy resources, especially fossil fuels, are still creating steeplechases to achieve the SDG's goal [5,29].

Ali et al. [18] have explored the three dimensions of sustainable development, i.e., economic, social, and environmental development, by approaching data from 2000 to 2021. A strong heterogeneity was observed in developed (G8) and developing (SAARC) nations by employing input-oriented data development analysis. A reasonable helping hand of developed nations (G8) toward developing nations (SAARC) can help to enhance global environmental sustainability and economic growth. Information and communication technologies (ICTs) deeply influence the environment positively or negatively [30,31]. It's another wide discussion. The negative impact of ICT is contributing as a CO_2_ emitter by producing ICT machinery, electric devices and recycling & reuses electronic waste with high energy consumption [32]. The positive and healthy contribution comes from the smarter cities establishment, well-structured electric grids, quick transportation systems, and accelerated industrial processes with fewer emissions. Higón et al. [20] confirmed an inverted U-shaped relationship between ICT and CO_2_ emission using data from 142 economies. Environmental improvement has increased with the development of the ICT sector in developed nations. Abid et al. [33] investigated that energy consumption increased due to the rapid increase in urbanization in G8 countries, which contemporarily became the reason for environmental degradation. This research focused on financial development and economic growth by approaching data from 1990 to 2019, while the FMOLS was used to check the periodical relationship among variables.

Shahbaz et al. [19] have checked the relationship between financial development, economic growth, and ecological footprints by considering non-renewable energy consumption and trade openness as additional factors. The research hit the ground of 10 countries (China, the USA, India, Japan, Brazil, Indonesia, Mexico, Korea, Turkey, and the UK) due to their high ecological footprints from 1992 to 2017. The Westerlund and Edgerton (2007) Panel LM bootstrap test confirmed the cointegration relationship among variables. A negative relationship between environmental quality with financial development, economic growth, and renewable energy consumption has been confirmed by the Common Correlated Effects (CCE) coefficient estimator [34,35]. Trade openness has shown an insignificant behavior toward ecological footprint. Financial development is unidirectional, while economic growth is bidirectionally interlinked with an ecological footprint [33,36]. The pollution haven [34,[37], [38], [39]] and halo [25,27,40] hypotheses are confirmed by previous literature. Usually, developed nations are considered the pollution halo hypothesis by transferring the highly concentrated and intensive polluted industries to developing nations.

The drastic change in global economic structure and trend of shifting manufacturing industries from developed to developing countries due to technological progress, economic reforms, and industrialization in East Asia, like China and India, is also observed. The pollution haven hypothesis has urged developed countries to shift the pollution-intensive industries from rigorous, environmentally regulated countries to loosely regulated countries via trade liberalization. Caglar et al. [27] have addressed the BRICS countries and their environmental situation by approaching their intensive consumption of natural resources, regular meetings tradition, and announcing policies to attain the Paris Agreement [31,41,42]. The non-linear panel autoregressive distribution lag technique explored the relationship among variables. The research findings support the mixture effect of natural resources (positive & negative) for short and long periods while the FDI behaved friendly toward environmental quality. The author also declared that the minimum consumption of fossil fuels could help to minimize CO_2_ emissions from energy resources. The load capacity factor of the top-10 economies stands on renewable energy and the effectiveness of competitive industrial performance (CIP) [43]. Using data from the top-10 economies from 1990 to 2018, Caglar & Askin [43] declared a negative impact of economic growth and competitive industrial performance on environmental sustainability using econometric panel techniques. The load capacity factor was improved by human capital and renewable energy consumption.

## 3 Data details and methodology

### 3.1 Data details

**Fig. 1:**
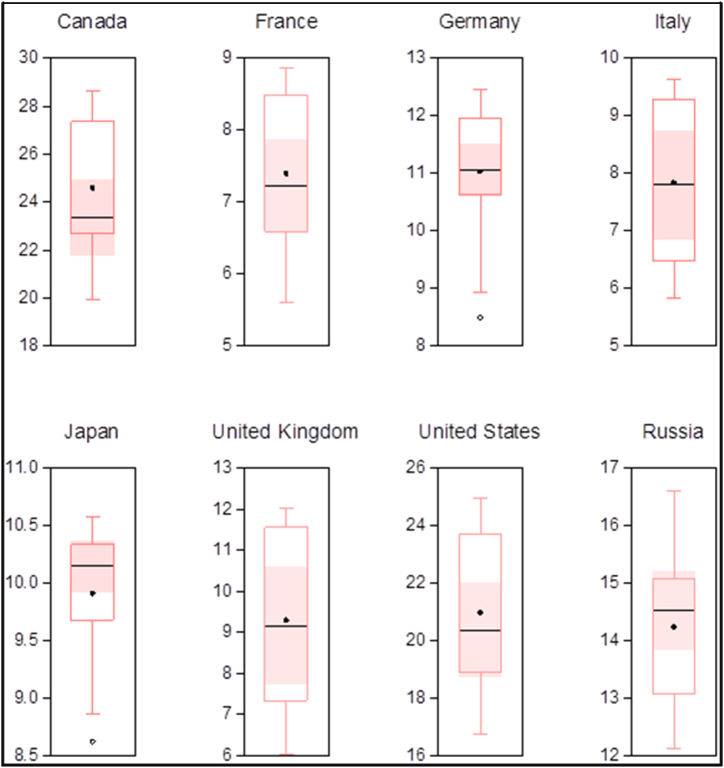
Per capita greenhouse gas emissions of individual country of G-8.

**Fig. 2:**
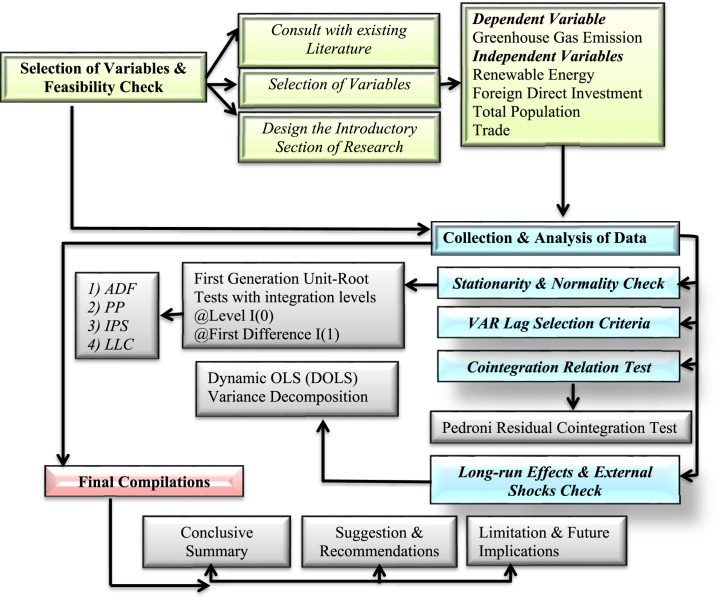
Research design & process.

This research will deal with annual time series data from 2000 to 2021 to determine the association of greenhouse gas emissions with renewable energy, foreign direct investment, total population, and trade. This research is related to G-8 countries listed as Canada, France, Germany, Italy, Japan, the United Kingdom, the United States, and Russia [18,19]. This research is based on secondary data and data mining of the selected set of variables has been done through the World Development Indicator [44]. The greenhouse gas emissions of G-8 countries are presented in figure-1. In table-1, the details of the individual variable are provided, and the variables’ abbreviations used throughout the research are mentioned right after the complete form of the variable (see Fig. 2).

**Table 1:** Description of variables.

Variables	Description
Greenhouse Gas Emission (GGE)	Total including LUCF (per capita)
Renewable Energy (RE)	Renewables per capita (kWh - equivalent)
Foreign Direct Investment (FDI)	FDI, net (BoP, current US$)
Total Population (TP)	Total, population
Trade	Percentage of GDP

### 3.2 Methodology

The main research model is presented in equation-1, which elaborates the division of exogenous and endogenous factors of research. The greenhouse gases are a dependent variable and demonstrate the environmental sustainability of G-8 countries.

(1) $$\begin{array}{c} GGE=f\left(RE,FDI,TP,TR\right)\\ {GGE}_{t}=\beta_{0}+\beta_{1}{RE}_{t}+\beta_{2}{FDI}_{t}+\beta_{3}{TP}_{t}+\beta_{4}{TR}_{t}+\varepsilon_{t} \end{array}$$

The whole analytical procession of this research will follow the main model and order of variables too. It is necessary to check the normality of the data series before starting the main correlation methods. The unit-root test $\left(y_{t}=y_{t-1}+\varepsilon_{t}\right)$ has explored the stochastic process of random walk of time series, which can create statistical interference problems. The unit-root test will help to check the normality and stationarity of data and identify the cointegration method. At the same time, the lag selection criterion finds a proper lag order for cointegration. The normality and stability of data series are checked by employing first-generation renowned unit-root tests to approach maximum accuracy rate and avoid ambiguities. The second econometric approach is used to narrow the VAR lag selection criteria, and the SIC-based lag is selected because of its accuracy for a small sample size. The VAR lag selection is essential for cointegration analysis and impulse response. The short-run and long-run relationship assumptions of variables and structural shocks are identified by VAR-based model impulse response.

(2) $$SIC\left(p\right)=\ln\left|\bar{\Sigma }\;\left(p\right)\right|+\frac{lnN}{N}\left(k^{2}p\right)$$

The Schwarz information criterion (SIC) based VAR lag selection model is presented above in equation-2, which signifies the deterministic regressor (seasonal dummies or intercept) [45,46]. The VAR epitomizes the true process of k-dimensional auto-regression of order p. As per the unit root and VAR lag selection tests, the Pedroni panel cointegration test was selected to check the cointegration relation among variables. The Pedroni cointegration test is designed to determine the null hypothesis of no cointegration in non-stationary data series. The advantageous feature of the Pedroni cointegration test is included common time dummies to accommodate the simple cross-sectional dependency by application of data time demeaning ${\bar{X}}_{t}=\frac{1}{N}\sum_{i=1}^{n}x_{i,t}$ for the individual variable. The test statistics are residual-based tests and collected by using equation-3.

(3) $$X_{i,t}=\alpha_{i}+\beta_{1i}y_{1i,t}+\beta_{2i}y_{2i,t}+\ldots +\beta_{ni}y_{ni,t}+\varepsilon_{i,t}$$

In equation-3, the term i is the number of individuals in the panel i=1,2,…,N, t is the time period t=1,2,…,t, and n is regressor number n=1,2,…,N. The credit for the original structuring and introduction of DOLS goes to Phillips and Hansen [47], who provided an optimal estimation of cointegration regression. The existence of a cointegration relationship was determined with the presence of serial correlation effects and endogeneity by the modified least squares method accurately. The Pedroni cointegration test determines the long-run relationship, and the coefficients of the cointegration equation are scrutinized by parametric approach of the dynamic ordinary least square (DMOLS) using the equational structure of equation-4.

(4) $${DOLS:X}_{t}=a+bY_{t}+\sum_{i=-k}^{i=k}\emptyset_{i}{\Delta Y}_{t+i}+\varepsilon_{t}$$

In equation-4, the term b is captured the elasticity for long-term and lag difference indicated by ∅ for I (1) regressor. The adjustment of non-normality residuals, endogeneity and autocorrelation as nuisance parameter has covered by coefficients [48,49]. The systematic evaluation of shocks reverberation has been done by variance decomposition econometric method. The total variance of the data set can be decomposed by attributing known (internal or treated) and unknown (external or error) sources. The variance of known variables is divided by unknown sources and compared to the theoretical F-ratio [50,51]. The comparatively greater value of the theoretical ratio indicates a statistically significant effect of known sources in generating total variance and contribution of individual variables in auto-regression, which can be calculated using equation-5.

(5) $$VD\;X_{it}=\frac{\Lambda_{i}Var(C_{t+k}-{\hat{C}}_{t+k/t}|\varepsilon_{t}^{MP})\Lambda_{i}^{\prime }}{\Lambda_{i}Var\left(C_{t+k}-{\hat{C}}_{t+k/t}\right)\;\Lambda_{i}^{\prime }}$$

In equation-5, the sign of $\Lambda_{i}$ denote the $i^{th}$ line of $\Lambda =\left(\Lambda^{f},\Lambda^{x}\right)$ and the standard decomposition ΛiVar(Ct+k−Cˆt+k/t|εtMP)Λi′ΛiVar(Ct+k−Cˆt+k/t)Λi′.

## 4 Empirical results

### 4.1 The unit-root test

The results of unit-root tests are displayed in table-2 with their significance levels. The asterisks placed at the top of coefficient values indicate significance at different levels. The first column of the table contains the nomenclature of unit-root tests. In contrast, the second row of the table includes the abbreviated information on the level of integration (LOI) and selected variables. The augmented-Dickey Fuller test has shown the positive significance of all variables at the first difference at 1%, and the constant of FDI is also significant at 1%. The results of the Phillip Perron test are pretty similar to ADF results. All variables are significant at 1% with the first difference, while FDI and trade are significant at a level with 1% & 5% significance levels, respectively. The LLC test confirms the significance of all variables at 1% with a negative sign as well as the significance of trade has been observed at a level with the same significance level & negative sign. The IPS test has shown the negative significance of all variables at 1% except TP. The TP is negatively significant at the 5% level. The results of the IPS test also confirmed the negative significance of FDI at 1% under I (0).

**Table 2:** Results of unit-root tests.

	LOI	GGE	RE	FDI	TP	TR
**ADF**	**I(0)**	2.365	2.508	34.225*	14.301	23.409
	**I(1)**	65.549*	55.184*	103.851*	36.854*	90.274*
**PP**	**I(0)**	2.049	11.346	64.832*	20.638	29.429**
	**I(1)**	137.483*	161.499*	306.529*	57.914*	131.615*
**LLC**	**I(0)**	0.713	2.552	−1.535	−0.433	−3.540*
	**I(1)**	−3.475*	−2.771*	−5.222*	−5.331*	−7.524*
**IPS**	**I(0)**	3.540	4.675	−2.839*	−0.133	−1.548
	**I(1)**	−6.017*	−5.004*	−9.412*	−1.569**	−7.779*

**Note**: The significance levels are displayed at the top of coefficients with asterisks *,** as 1% & 5% level.

### 4.2 VAR lag order selection criteria

The second step of analysis is lag order selection criteria which indicate the obvious way optimal lag selection after the determination of the initial VAR model and the results of it are presented in table-3. The lags are a lap of time that comes into existence with respect to dependent variables’ responses to independent variables. The Schwartz criterion is considered good-to-fit for this research because the economists prefer that the SIC criterion is best when the study deals with more than 3 variables [45,46]. The SIC criterion shows significance at 1% and selected lag 1. The sequential search of lag length refers to the charge of data mining [52]. The incorrect lag specification will lead to the misspecification of errors.

**Table 3:** VAR lag order selection criteria.

Lag	LogL	LR	FPE	AIC	SIC	HQ
0	−7042.971	NA	3.14 × 10^48^	125.857	125.978	125.906
1	−5779.029	2392.461	7.73 × 10^38^	103.733	104.461*	104.028
2	−5725.722	96.143	4.68 × 10^38^	103.227	104.562	103.769
3	−5683.787	71.889*	3.48 × 10^38^*	102.925*	104.867	103.713*
4	−5665.618	29.524	3.98 × 10^38^	103.047	105.595	104.081
5	−5649.027	25.480	4.72 × 10^38^	103.197	106.352	104.477
6	−5628.849	29.186	5.31 × 10^38^	103.283	107.045	104.810

**Note:** The automated selected lag under a specific criterion is indicated by placing an asterisk at the coefficient value. The capital “E” is used for exponential power.

### 4.3 Cointegration test

The results of the Pedroni cointegration approach are covered in table-4. The cointegration approach usually identifies the long-run relationship among selected variables, and the cointegration model selection is based on the unit-root test results. The specialty of the Pedroni cointegration technique and the reason behind its choice is an exploration of two test-statistics classes, i.e., panel statistics and group mean statistics. The panel statistics performs its role to equivalent the unit-root statistics against homogeneity while the group-mean statistics is analogous to the panel unit-root test against heterogeneity. The Pedroni is a panel and intra-panel cointegration technique. The results within-dimension confirmed the negative significance of panel PP & ADF at 1% & 5% for statistics and weighted statistic while a similar result of between-dimension for group PP & ADF at 5% & 10% have observed.

**Table 4:** Pedroni residual cointegration test.

Automatic lag length selection based on SIC with a max lag of 3				
Newey-West automatic bandwidth selection and Bartlett kernel				
Alternative hypothesis: common AR coefficients				
Within-Dimension	Statistic	Weighted Statistic	Between- Dimension	Statistic
**Panel rho**	−0.077	0.139	**Group rho**	1.404
	0.469	0.555		0.920
**Panel PP**	−2.621*	−2.278**	**Group PP**	−1.756**
	0.004	0.011		0.040
**Panel ADF**	−2.544*	−2.229**	**Group ADF**	−1.510***
	0.006	0.013		0.066

**Note:** The significance levels are displayed at the top of coefficients with asterisks *, **, ** as 1%, 5% and 10% level.

### 4.4 The dynamic ordinary least square (DOLS)

The results of DOLS are precisely and perfectly placed in table-5, which helps to recheck the accuracy of coefficients' cointegration relation estimation simply and efficiently [48,49]. The long-run relationship of FDI, TP and RE has been confirmed with different signs of significance. These signs are gesticulations that decide the impact of independent variables on the dependent variable. A negative relationship between renewable energy (−0.004*) and FDI (−1.61* × 10^−11^) with GHG is confirmed [16,17]. Total population (8.68* × 10^−6^) and trade (0.316*) are positively related to GHG emissions [8,53,54]. Total population and trade increased the level of GHG emissions in G-8 countries, while the other variables' negative signs confirmed their declining contribution toward GHG emissions [55]. The results of DOLS confirmed that GHG emissions' growth speed is higher than economies’ sectorial growth. To achieve net-zero carbon, G-8 countries should bring up the scale of sectorial growth parallel to GHG emissions growth first and then change the scale oppositely, i.e. lower GHG emissions and higher sectorial growth. The Total population and trade are contributed positively to GHG emissions which can be reduced with green technological-based products and public awareness about environmentally friendly gadgets.

**Table 5:** Panel dynamic ordinary least squares (DOLS).

Fixed leads and lags specification (lead = 1, lag = 1)			
Long-run variance weights (Bartlett kernel, Newey-West fixed bandwidth)			
Variable	Coefficient	Std. Error	Prob.
**RE**	−0.004*	6.86 × 10^−10^	0
**FDI**	−1.61* × 10^−11^	1.07 × 10^−17^	0
**TP**	8.68* × 10^−6^	3.41 × 10^−12^	0
**TR**	0.316*	3.82 × 10^−8^	0

**Note:** The significance levels are displayed at the top of coefficients with asterisks *,** as 1% & 5% level. The capital “E” is used for exponential power.

### 4.5 Variance decomposition

The variance decomposition implied checking the contribution of individual variables to the other with the specification of auto-regression [50,51]. The variance decomposition results are presented in table-6, which determines the forecasting error variance of a particular variable to be explained by exogenous shocks. The variance decomposition is quite essential to measure the shocks. By utilizing the order of Cholesky decomposition suggested by Sim [56], variance decomposition results follow the order of orthogonalizing innovations [57,58] in the vector auto-regression (VAR) equations, i.e., this research explored 1–10 periods horizontally presented in the table. The results indicated that greenhouse gas emissions respond to shock all variables with inclining and declining perspectives. Their shocks explain more than 87% portion of GHG emissions. The rest of the variables influencing the percentage of shocks are RE 0.613%, FDI 0.028%, TP 19.404%, and TR 7.129% [59,60].

**Table 6:** Variance decomposition of per capita greenhouse gas emission.

Period	S.E.	lnGHG	lnRE	lnFDI	lnTP	lnTR
1	0.438	100.000	0.000	0.000	0.000	0.000
2	0.607	93.904	0.556	0.115	0.987	4.437
3	0.752	90.628	0.625	0.087	3.127	5.533
4	0.886	87.501	0.668	0.068	5.659	6.105
5	1.009	84.501	0.681	0.057	8.315	6.447
6	1.124	81.717	0.679	0.048	10.898	6.658
7	1.232	79.159	0.669	0.040	13.325	6.807
8	1.333	76.832	0.653	0.034	15.556	6.926
9	1.427	74.725	0.634	0.030	17.581	7.030
10	1.515	72.826	0.613	0.028	19.404	7.129

### 4.6 Discussion

The importance of environmental sustainability is realized globally, and the focus of countries, if not fully moved from development to ecological sustainability, so at maximum extent, it is going happen. Foreign direct investment is based on contribution to environmentally friendly projects. The assessments of development projects are keenly focused on the impact of the environment these days. Researchers and environmentalists are urging the world to replace the polluted resources with environmentally friendly development equipment and resources because of the rapid climate change and harmful ultraviolet rays enhancing global warming. This research is also conducted to determine the GHG emissions and sectorial growth of G-8 countries. The research data is approached from 2000 to 2021 for GHG emissions, renewable energy, foreign direct investment, total population, and trade. The econometric techniques are used to check the normality and stationarity of data series and measure the cointegration regression. The dynamic ordinary least square was employed to recheck the cointegration relationship among variables. At the same time, the variance decomposition analysis determines the effects of external shocks on the fluctuality or intensity of GHG emissions. Renewable energy and foreign direct investment have confirmed a significant negative relationship with GHG emissions, indicating its sustainable environmental modernity and ecological clamour. G-8 countries are following the Paris Agreement to achieve net zero emissions and turn the financial directory toward green finance, which positively affects GHG emissions reduction.

## 5 Conclusion

This research insightfully focused on designing policies to sustain the climate situation for habitats with the growth of leading economic sectors. The positive contribution of RE and FDI to GHG emissions is too apparent and direct, which covers the hydrosphere, lithosphere, biosphere and atmosphere via energy resources and FDI practices. The long-term negative contribution of the total population and trade in GHG emissions confirmed that the green technological process and increase in renewable energy reduce the intensity of GHG emissions. The global contribution of G-8 countries in economic development and GHG emissions cannot deny. Usually, researchers and environmentalists ignore this group of countries due to its sustained and slower GHG emissions growth. In contrast, detecting the exact sector of toxic emissions can reduce emissions. As per this research, the population and trade activities of G-8 countries enhance the burden of GHG emissions which can be cut down by adopting green sectorial development methods. Population and trade have increased GHG emissions because the people of G-8 countries are not following green practices in daily life.

### 5.1 Limitations, recommendations & future implications

The awareness program, workshops, street messages and international conferences can promote environmentally friendly activities and reduce the impact of the population increase in GHG emissions. This research pointed out a very insightful point: the impact of FDI and trade. FDI decrease GHG emission and trade increases it because the FDI is based on environmental practices these days. The investors confirmed the environmental impact of investment with profitability that's why the FDI is supervised meanwhile the trading activities are not checked with keen concern. G-8 countries should discourage environmentally destructive trade methods and observe the manufacturing sector keenly. The industrial and manufacturing sector should embolden the adaptation of cleaner production methods according to global environmental regulations. Usually, the low-income countries' FDI participates in environmental degradation and supports the pollution haven hypothesis (PHH). G-8 countries are developed and well-structured, so they should provide extra incentives to environmentally friendly industries and cancel the license of pollution-intensive industries. The industrial and manufacturing industries with medium pollution-intensive should operate with specific measures and operational permissions and help replace their technical part with green technology with the implementation of environmental regulations. Foreign direct investment and trade should be keenly observed and reassured of the implementation of environmental protection rules.

## Author contribution statement

Sobia Naseem: Conceived and designed the experiments; Performed the experiments; Wrote the paper.

Xuhua HU: Analyzed and interpreted the data.

Muhammad Mohsin: Analyzed and interpreted the data; Contributed reagents, materials, analysis tools or data.

## Data availability statement

Data associated with this study has been deposited at World Development Indicator (WDI); Our World in Data (OWD) https://databank.worldbank.org/source/world-development-indicators#; https://ourworldindata.org/

## Ethical approval

Not Applicable.

## Funding

This research is funded by Jiangsu Funding Program for Excellent Postdoctoral Talent under funding number [2022ZB661].

## Declaration of competing interest

The authors declare that they have no known competing financial interests or personal relationships that could have appeared to influence the work reported in this paper.
